# Enhancing Oral Squamous Cell Carcinoma Detection Using Histopathological Images: A Deep Feature Fusion and Improved Haris Hawks Optimization-Based Framework

**DOI:** 10.3390/bioengineering11090913

**Published:** 2024-09-12

**Authors:** Amad Zafar, Majdi Khalid, Majed Farrash, Thamir M. Qadah, Hassan Fareed M. Lahza, Seong-Han Kim

**Affiliations:** 1 Department of Artificial Intelligence and Robotics, Sejong University, Seoul 05006, Republic of Korea; amad@sejong.ac.kr; 2 Department of Computer Science and Artificial Intelligence, College of Computing, Umm Al-Qura University, Makkah 24382, Saudi Arabia; mknfiai@uqu.edu.sa (M.K.); mmfarrash@uqu.edu.sa (M.F.); 3 Department of Computer and Network Engineering, College of Computing, Umm Al-Qura University, Makkah 24382, Saudi Arabia; tmqadah@uqu.edu.sa; 4 Department of Cybersecurity, College of Computing, Umm Al-Qura University, Makkah 24382, Saudi Arabia; hflahza@uqu.edu.sa

**Keywords:** oral squamous cell carcinoma, machine learning, oral cancer, mouth cancer

## Abstract

Oral cancer, also known as oral squamous cell carcinoma (OSCC), is one of the most prevalent types of cancer and caused 177,757 deaths worldwide in 2020, as reported by the World Health Organization. Early detection and identification of OSCC are highly correlated with survival rates. Therefore, this study presents an automatic image-processing-based machine learning approach for OSCC detection. Histopathological images were used to compute deep features using various pretrained models. Based on the classification performance, the best features (ResNet-101 and EfficientNet-b0) were merged using the canonical correlation feature fusion approach, resulting in an enhanced classification performance. Additionally, the binary-improved Haris Hawks optimization (b-IHHO) algorithm was used to eliminate redundant features and further enhance the classification performance, leading to a high classification rate of 97.78% for OSCC. The b-IHHO trained the k-nearest neighbors model with an average feature vector size of only 899. A comparison with other wrapper-based feature selection approaches showed that the b-IHHO results were statistically more stable, reliable, and significant (*p* < 0.01). Moreover, comparisons with those other state-of-the-art (SOTA) approaches indicated that the b-IHHO model offered better results, suggesting that the proposed framework may be applicable in clinical settings to aid doctors in OSCC detection.

## 1. Introduction

Mouth cancer, including oral squamous cell carcinoma (OSCC), is one of the most prevalent and fatal diseases and has long been a significant public health concern worldwide [1]. OSCC is a well-known malignant tumor with a high incidence rate, with an estimated 1,401,931 cases reported globally in 2019 [2,3]. As of 2018, OSCC accounted for approximately 25% of upper aerodigestive tract cancer cases in France [4], ranking sixth in terms of cancer frequency. Although betel quid chewing, tobacco use, and excessive alcohol consumption are the primary risk factors for OSCC [5], individuals that do not participate in these activities, especially those aged <45 years, can still develop this malignancy. According to a previous study [6], young women with tongue cancer are at a high risk of developing OSCC.

In 2020, OSCC had a high annual mortality rate of 170,000, primarily because of late-stage detection [4]. According to a 2008 study [7], OSCC is primarily diagnosed in men and women with median ages of 61.5 and 66.4 years, respectively. Another study published in 2022 reported that the average age of patients with OSCC is 62 years [8]. The cancer stage at diagnosis is highly correlated with survival rates. Early screening and detection are essential for improving patient prognosis, and blood tests and visual examinations are conventionally employed for detecting OSCC.

However, clinical diagnoses are more successful for detecting cancer in the oral cavity, primarily through X-rays [9], computed tomography (CT) scans [10], positron emission tomography (PET) scans [11], magnetic resonance imaging (MRI) [12], and endoscopies [13]. The biopsy procedure involves removing tiny tissue samples from the oral region, including malignant tumors. X-rays are frequently used to track changes in the teeth and jawbone anatomies, which aids in detecting any malignant growths that metastasize to the jawbone tissue. CT scans combine several X-rays obtained from various angles to produce intricate 3D images of the mouth and throat, allowing medical professionals to spot anomalies or indications of malignant growths. Additionally, PET scans involve injecting a small quantity of radioactive material into the body and using a specialized camera to detect the material, whereas MRI scans offer a precise visualization of the oral cavity and adjacent structures. This is beneficial for identifying malignancies located deep within tissues. Furthermore, endoscopy is particularly useful for inspecting the throat and larynx and identifying cancers in difficult-to-reach locations. Fluorescence endoscopy can be used to visualize oral malignancies on the outer surfaces that are difficult to detect using white light. However, physical examinations can be expensive, time consuming, and require specialized knowledge for accurate result interpretations. 

The rapidly evolving field of artificial intelligence comprises novel diagnostic techniques that can potentially facilitate early OSCC detection and classification [14], which are essential for providing timely and optimal treatment [15]. Several machine and deep learning models have been developed for this purpose [16]. Amin et al. [17] employed three pretrained models, namely InceptionV3, VGG16, and ResNet50, to extract features from histopathology images and merged them to form a new feature vector, achieving a high accuracy of 96.55% compared to individual models. Subsequently, Das et al. [18] developed a deep learning model that obtained better results (97.5% accuracy) than pretrained models. In 2023, Das et al. [19] designed the simplest 10-layer deep convolutional neural network (CNN) architecture to detect OSCC from histopathological images and obtained promising results. In another study [20], a framework comprising the MobileNet-V2 and Darknet-19 models was used to extract deep features, and traditional machine learning classifiers were used for classification, resulting in an accuracy of 92%. It employed a serial-based feature fusion approach for feature concatenation, and chaotic crow search optimization for optimal feature selection, resulting in a high computational complexity. 

In conclusion, the aforementioned studies focused on developing deep learning and pretrained models for OSCC detection. However, they did not modify the layer structure or employ hyperparameter optimization, model selection, information fusion, and optimal feature selection to lower the computational time and increase accuracy. 

Therefore, this study presents an automatic machine learning-based approach for OSCC detection using histopathological images, wherein pretrained models were used to obtain the deep features from the acquired images. Based on the classification performance, deep features were merged using the canonical correlation feature fusion approach. Subsequently, various wrapper-based approaches were tested to remove redundant features and enhance the classification accuracy. Finally, based on the results, the binary-improved Haris Hawks optimization (b-IHHO) was used to further enhance the classification performance and reduce the feature vector size. The k-nearest neighbors (KNN) algorithm was used to evaluate the classification performance of the proposed framework. Its results were compared with those of other wrapper-based feature selection approaches, and a *t*-test was conducted to determine their statistical significance. Additionally, the results were compared with those obtained using other SOTA approaches.

## 2. Materials and Methods

### 2.1. Proposed OSCC Detection Framework

Histopathological imaging is a reliable method for detecting and diagnosing OSCC. Typically, histopathological images are obtained from tissue samples and examined under a microscope to detect the presence of abnormal cells, providing additional information to guide subsequent investigations and treatments to achieve better outcomes.

**Figure 1:**
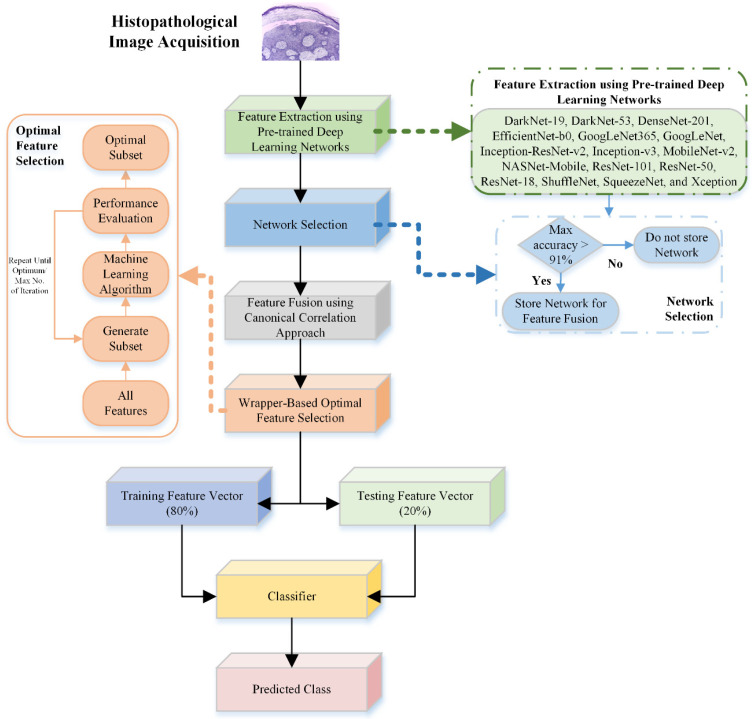
Flowchart of the proposed OSCC-detection framework using histopathological images.

This study presents an automatic detection and identification machine learning framework for OSCC. After acquiring the OSCC images, deep features were extracted using various pretrained deep learning models. Next, the deep features extracted from models with classification accuracies greater than 91% were fused using a canonical correlation approach. Subsequently, wrapper-based optimal feature selection approaches were employed to further enhance the classification performance. A flowchart of the proposed OSCC detection approach is shown in Figure 1.

### 2.2. Histopathological Images Dataset

This study employed the online biopsy dataset Histopathologic Oral Cancer Detection using CNNs (https://www.kaggle.com/datasets/ashenafifasilkebede/dataset; accessed 13 July 2024). Medical experts collected, prepared, and categorized the slides comprising H&E-stained tissues of 230 patients using a Leica ICC50 HD microscope(Leica Microsystems, Wetzlar, Germany). The details and examples of images in these datasets are presented in Table 1.

### 2.3. Feature Extraction from Histopathological Images

#### 2.3.1. CNNs

CNNs, also known as ConvNETs, are a subclass of artificial neural networks that handle data in a grid-like layout. They can be used to identify various features in an image, such as corners and edges, and effectively eliminate the need for handcrafted feature extraction approaches by including them in their architecture. They comprise various layers, such as input convolution, rectified linear unit (ReLU), and pooling, for extracting image features and information. Finally, a fully connected layer retrieves features for image classification [21,22]. The other fundamental elements of CNNs are the weights, neurons, bias factors, and activation functions.

#### 2.3.2. Deep Feature Extraction Using CNNs

**Figure 2:**
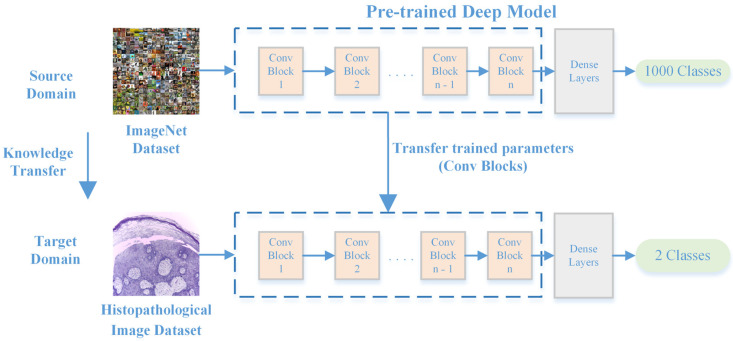
Modified AlexNet for deep feature extraction through transfer learning.

The performance of a CNN can be improved by using a larger training dataset. Transfer learning is a process that allows transferring knowledge from one domain to another. It involves transferring knowledge from a model trained for solving a particular problem and reusing it to solve another related problem. In this study, we assumed a domain with two components [23,24]:

(1) $$d^{m}=A+prob\left(a\right),$$

where A and prob(a) denote the feature space and marginal probability, respectively. We assume that a task has the following elements:

(2) $$t^{r}=B+\omega ,$$

where B and ω are space and objective functions, respectively. Additionally, let ${d_{s}}^{m}$ and ${t_{s}}^{r}$ denote the source domain and the task, respectively, and ${d_{t}}^{m}$ and ${t_{t}}^{r}$ denote the target domains and tasks, respectively. In transfer learning, the source information is used to learn the conditional probabilities of the target domain. Several pretrained models have been developed for various medical imaging applications [25,26]. Figure 2 shows the basic transfer learning concept employed by AlexNet for deep feature extraction from histopathological images in ImageNet.

In this study, various pretrained deep learning models, such as Xception, SqueezeNet, ShuffleNet, ResNet-18, ResNet-50, ResNet-101, NASNet-Mobile, MobileNet-v2, Inception-v3, Inception-ResNet-v2, GoogLeNet, GoogLeNet365, EfficientNet-b0, DenseNet-201, DarkNet-53, and DarkNet-19, were used for deep feature extraction from histopathological images for OSCC detection.

#### 2.3.3. Feature Fusion Using Canonical Correlation Analysis

This study employed a canonical correlation analysis approach to fuse the deep features acquired from histopathological images. The basic principle of the canonical correlation analysis approach is to maximize the correlation between two features. Assuming that there are two feature sets ($f_{x}\in R_{a_{1}\times b}$ and $f_{y}\in R_{a_{2}\times b}$) with *n* features, where $a_{1}$ and $a_{2}$ denote feature dimensions, they can be defined as

(3) $$\left.\begin{array}{c} f_{x}=\left[f_{x}^{1},f_{x}^{1},...,f_{x}^{n}\right]\\ f_{y}=\left[f_{y}^{1},f_{y}^{1},...,f_{y}^{n}\right] \end{array}\right\}.$$

A linear transfer function for the above equation can be defined as

(4) $$\sigma =\max\left(W_{x},W_{y}\right)\left(\frac{W_{x}^{T}C_{xy}W_{y}}{\left(W_{x}^{T}C_{xx}W_{x}\right)\left(W_{y}^{T}C_{yy}W_{y}\right)}\right).$$

Additionally, within-covariance matrices can be defined as $C_{xx}\in R^{a_{1}\times a_{1}}$ or $C_{xy}\in R^{a_{1}\times a_{2}}$, where $C_{xx}\in R^{a_{1}\times a_{1}}$ denotes the feature set covariance matrix. Hence, the canonical correlation analysis approach can be defined as

(5) $$\left.\begin{array}{c} {C_{xx}}^{-1}C_{xy}{C_{yy}}^{-1}C_{yx}W_{x}=\sigma W_{x}\\ {C_{yy}}^{-1}C_{yx}{C_{xx}}^{-1}C_{xy}W_{y}=\sigma W_{y} \end{array}\right\}.$$

The following equation can be used to compute the final transformed fused vector:

(6) $$\tilde{Z}={W_{x}}^{T}\sigma_{x,i}+{W_{y}}^{T}\sigma_{y,i}={W_{x}}^{T}{W_{y}}^{T}\left[\begin{array}{c} \sigma_{x,i}\\ \sigma_{y,i} \end{array}\right].$$

#### 2.3.4. HHO

HHO is a computationally intelligent approach that replicates predator–prey interaction patterns of Harris hawks [27]. It comprises three primary stages: exploration, transformation, and exploitation. HHO has obtained promising results in mining applications owing to its efficient global search capability and minimal parameter adjustments. It employs the following methods to locate prey in diverse locations:

(7) $$M\left(t+1\right)=\left\{\begin{array}{cc} M_{r}(t)-r_{a}\left|M_{r}(t)-2r_{b}M(t)\right|, & q\geq 0.5\\ \left|M_{rab}(t)-M_{avg}(t)\right|-r_{c}\left[L+r_{d}\left(u-l\right)\right], & q<0.5 \end{array},\right.$$

where M(t), $M_{rab}(t)$, $M_{r}(t)$, and $M_{avg}(t)$ denote the current, rabbit, random, and average positions of the hawks at t, respectively, whereas r is a random value between 0 and 1. Additionally, u and l represent the lower and upper boundaries, respectively. $M_{avg}(t)$ is calculated as follows:

(8) $$M_{avg}\left(t\right)=\sum_{n=1}^{N}\frac{M_{n}\left(t\right)}{N},$$

where N and $M_{n}(t)$ denote the population size and the position of the *n*th individual, respectively. Depending on the prey’s energy ($E_{energy}$) (defined in Equation (9)), HHO switches between searching and various developmental actions.

(9) $$E_{energy}=2\times E_{energy,o}\left(1-\frac{t}{s}\right),$$

where $E_{energy,o}$ is a random value between −1 and 1, s is the maximum number of iterations, and t is the current iteration. If $\left|E_{energy}\right|$ > 1, it enters the development phase; otherwise, it remains in the search space. Soft and hard besieges occur depending on the conditions for updating the position, which are obtained as follows:

(10) $$M(t+1)=\left\{\begin{array}{cc} \left(M_{rab}(t)-M(t)\right)-E_{energy}\left|2\left(1-r_{e}\right)M_{rab}(t)-M(t)\right|, & 0.5\leq \left|E_{energy}\right|<1\;and\;r_{e}\geq 0.5\\ M_{rab}(t)-E_{energy}\left|M_{rab}(t)-M(t)\right|, & \left|E_{energy}\right|<0.5\;and\;r_{e}\geq 0.5 \end{array},\right.$$

where $r_{e}$ and M(t) denote a random number and the current prey position, respectively. When $0.5\leq \left|E_{energy}\right|<1\;and\;r_{e}<0.5$, the algorithm uses the following equations to update the position (soft besiege progressive rapid dives approach):

(11) $$M(t+1)=\left\{\begin{array}{cc} A, & f(A)<f(M(t))\\ B, & f(B)<f(M(t)) \end{array},\right.$$

(12) $$A=M_{rab}\left(t\right)-E_{energy}\left|2\left(1-r_{e}\right)M_{rab}\left(t\right)-M\left(t\right)\right|,$$

(13) $$B=A+r_{and}\left(d_{im}\right)\times l_{evy}\left(d_{im}\right),$$

where f(*), $r_{and}$, and $l_{evy}$ denote the fitness function, random vector size of the problem dimension ($d_{im}$), and Levi’s flight, respectively. When $\left|E_{energy}\right|<0.5\;and\;r_{e}<0.5$, the algorithm uses the following equations to update the position (hard besiege progressive rapid dives approach):

(14) $$M(t+1)=\left\{\begin{array}{cc} A, & f(A)<f(M(t))\\ B, & f(B)<f(M(t)) \end{array},\right.$$

(15) $$A=M_{rab}\left(t\right)-E_{energy}\left|2\left(1-r_{e}\right)M_{rab}\left(t\right)-M_{avg}\left(t\right)\right|,$$

(16) $$B=A+r_{and}\left(d_{im}\right)\times l_{evy}\left(d_{im}\right).$$

#### 2.3.5. IHHO

In 2023, Peng et al. [28] presented an improved version of the HHO to enhance the individual linkages between the populations of the HHO such that individuals with better fitness values take the lead and influence the remaining population to adjust their positions. Herein, the individuals are ranked according to their fitness values and denoted as α, β, and γ, respectively. Individual α is the first step, and its position-update formula is obtained as follows: When the ratio of the remaining running times of the algorithm to the total running times with the Cauchy random number is compared, the current position has a certain probability of moving closer to the optimal position, whereas the later-stage replacement probability is more negligible, which effectively ensures that the algorithm does not fall into local optimization.

(17) $$M^{i}(t+1)=\left\{\begin{array}{cc} M_{rab}^{i}(t), & \left(\tan\left(\pi (rand-0.5)\right)<\left(1-\frac{t}{s}\right)\right)\\ M_{rab}^{i}(t)+\left(4-t\times \left(\frac{4}{s}\right)\right)\times rand\left(M_{m}^{i}(t)-M_{n}^{i}(t)\right), & other \end{array}\right.$$

where $M_{m}^{i}(t)$ and $M_{n}^{i}(t)$ are randomly selected individuals from the population that do not belong to α and i represents the dimensions. The following equations present the position-update formulas for individuals β and γ, respectively. Both $M_{e}$ and $M_{f}$ were randomly selected.

(18) $$M^{i}(t+1)=\left\{\begin{array}{cc} M^{i}(t), & rand>0.5\\ \frac{M_{\alpha }^{i}(t)+M_{\beta }^{i}(t)}{2}, & other \end{array},\right.$$

(19) $$M^{i}\left(t+1\right)=\left\{\begin{array}{cc} \frac{M_{e}^{i}\left(t\right)+X_{f}^{i}\left(t\right)}{2}, & rand>0.5\\ \frac{\left(M_{\alpha }^{i}\left(t\right)+M_{\beta }^{i}\left(t\right)+M_{\gamma }^{i}\left(t\right)\right)}{3}, & other \end{array}.\right.$$

**Figure 3:**
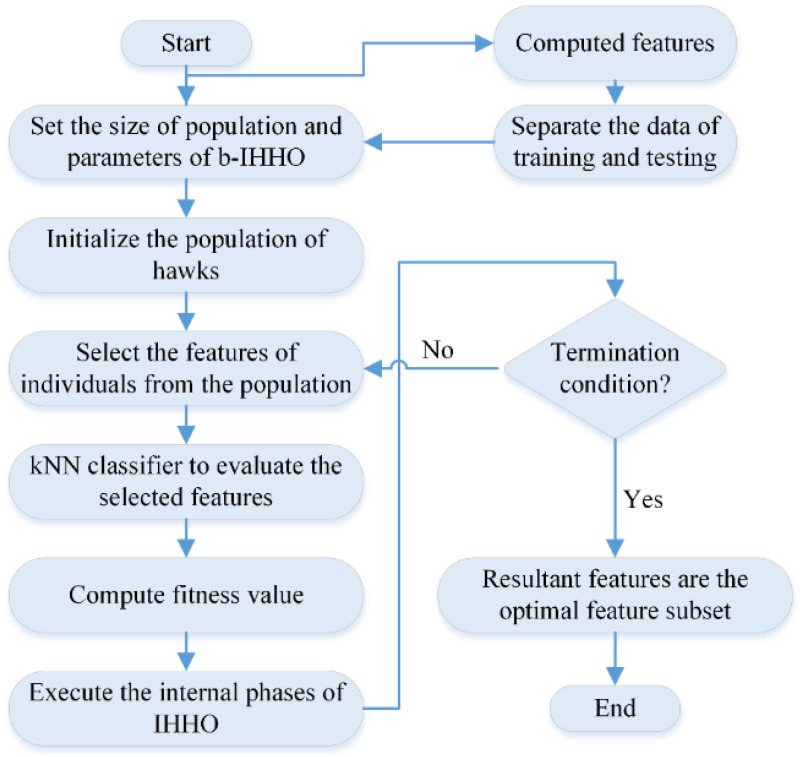
b-IHHO flowchart for selecting optimal deep features.

Three excellent individuals (α, β, and γ) are involved in local development, whereas the others contribute to the original HHO updates. Individuals’ optimal locations were awarded in decreasing order, which encourages localized growth within the field being investigated. Individuals β and γ are connected to α, which improves the communication between outstanding individuals. Compared to the original HHO, the updating technique for these three individuals is relatively simple. This method, known as IHHO, enables quicker execution and higher accuracy for feature selection tasks, while reducing temporal complexity [28]. Additionally, IHHO is converted into a discrete optimization problem, known as b-IHHO, for feature selection. Further details regarding b-IHHO can be found in [28], and the b-IHHO flowchart for selecting the optimal features is illustrated in Figure 3.

Finally, the KNN is used as a classifier to evaluate the selected features using the following fitness function:

(20) $$Fitness\;function\;\left(J\right)=\sigma \left(1-\frac{Correctly\;classified\;images}{Total\;no.of\;images}\right)+\left(1-\sigma \right)\left(\frac{f_{SL}}{f_{FL}}\right),$$

where σ, $f_{SL}$, and $f_{FL}$ denote the weight factor, number of features selected, and total number of features, respectively. The value of σ is 0.99 [29].

## 3. Results

In this study, various pretrained deep learning models, such as Xception, SqueezeNet, ShuffleNet, ResNet-18, ResNet-50, ResNet-101, NASNet-Mobile, MobileNet-v2, Inception-v3, Inception-ResNet-v2, GoogLeNet, GoogLeNet365, EfficientNet-b0, DenseNet-201, DarkNet-53, and DarkNet-19, were used for deep feature extraction from histopathological images for OSCC detection. MATLAB 2023b was used for processing, running on a PC with the following specifications: 12th Generation Intel(R) Core (TM) i7 CPU, 1 TB SSD, NVIDIA GeForce RTX 3050 GPU, 32 GB of RAM, and 64-bit Windows 11. Additionally, the 0.2 holdout validation approach is used to train and test the models. 

First, all the pretrained models were used to extract the deep features before applying the softmax layer. The KNN models were trained using the deep features extracted using each model; the results are presented in Table 2. 

**Figure 4:**
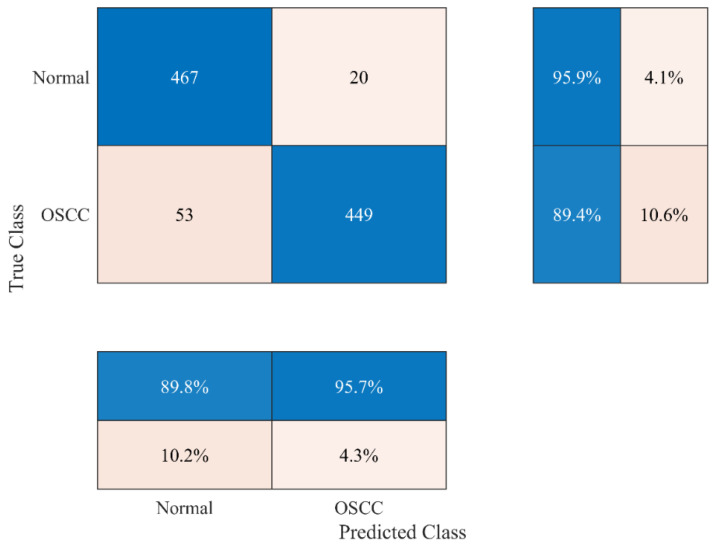
Confusion matrix for OSCC detection obtained by applying the canonical correlation feature fusion approach.

A comprehensive analysis of the results revealed that the deep features extracted using the ResNet-101 and EfficientNet-b0 models, which had feature vector sizes of 2048 and 1280, respectively, yielded the highest accuracies of 91.51% and 91.61%, respectively. The canonical correlation feature fusion approach was applied to enhance the classification performance and reduce the feature vector size, as discussed in Section 2.3.3. The results are presented in Figure 4.

The result in Figure 4 indicates that the canonical correlation feature fusion approach further enhanced the classification accuracy to 92.62%, with a feature vector size of 2560. This is because it removes redundant features and fuses them to form a new feature training vector.

**Figure 5:**
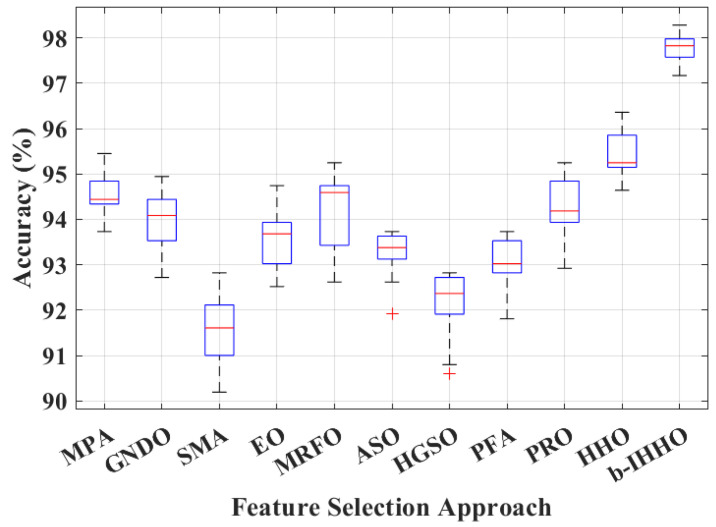
Classification performances of various wrapper-based approaches for OSCC detection with b-IHHO over ten runs (*p* < 0.01). MPA: marine predator algorithm, GNDO: generalized normal distribution optimization, SMA: slime mold algorithm, EO: equilibrium optimizer, MRFO: manta ray foraging optimization, ASO: atom search optimization, HGSO: Henry gas solubility optimization, PFA: pathfinder algorithm, PRO: poor and rich optimization, HHO: Harris hawks optimization, and b-IHHO: binary improved HHO.

To further enhance the classification performance for OSCC detection, various wrapper-based optimal feature selection approaches, such as the marine predator algorithm, generalized normal distribution optimization, slime mold algorithm, equilibrium optimizer, manta ray foraging optimization, atom search optimization, Henry gas solubility optimization, pathfinder algorithm, poor and rich optimization, HHO, and b-IHHO, were employed. The results are presented in Figure 5 using a box-whisker plot for ten runs. 

In wrapper-based approaches, the extracted optimal features tested using a machine learning classifier (k-NN) guarantee high reliability and better classification accuracy. Figure 5 shows the classification performance enhancement for all the wrapper-based optimal feature selection approaches. Each algorithm was run ten times, and the results are presented as box-whisker plots. A careful analysis of the results revealed that HHO exhibited the best classification performance. Therefore, the advanced HHO (b-IHHO) variant was employed to further enhance the classification accuracy to 98.28% (mean = 97.78%), as shown in Figure 5. Specifically, b-IHHO resulted in an average increase of 2.32% in the classification performance compared to the simple HHO. 

**Figure 6:**
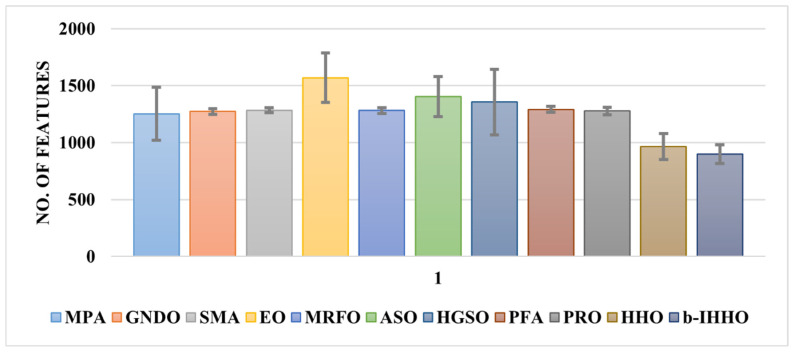
Optimal number of features for each wrapper-based method (average ± standard deviation). MPA: marine predator algorithm, GNDO: generalized normal distribution optimization, SMA: slime mold algorithm, EO: equilibrium optimizer, MRFO: manta ray foraging optimization, ASO: atom search optimization, HGSO: Henry gas solubility optimization, PFA: pathfinder algorithm, PRO: poor and rich optimization, HHO: Harris hawks optimization, and b-IHHO: binary improved HHO.

Subsequently, a two-sample *t*-test was employed to prove the statistical significance and reliability of the results, which were highly accurate (*p* < 0.01, 99% confidence interval), as demonstrated by the *t*-test. Furthermore, Cohen’s d value of −5.24 was obtained for the effect sizes of the HHO and b-IHHO results. This value indicates a large effect size, suggesting a significant difference between the two approaches. Additionally, the negative sign indicates that the mean HHO accuracy was lower than the mean accuracy of b-IHHO. The average numbers of features used to train the models are shown in Figure 6. 

The results in Figure 6 indicate that each algorithm removed a considerable number of redundant features compared to the fused feature vector (2560) and b-IHHO employed fewer features on average for training the KNN model to obtain a high classification accuracy.

## 4. Discussion

Various protocols can help doctors detect and diagnose OSCC. One crucial aspect is the detailed examination of histopathological biopsy images, which helps us to understand the disease progression and stage, enabling appropriate and timely treatment. However, a highly skilled pathologist is required to distinguish between healthy and cancerous cells in oral biopsy images. However, this process is time consuming, leading to delayed detection and treatment. Therefore, an automated OSCC detection approach is required for faster and more accurate OSCC diagnosis. 

The automatic OSCC detection approach proposed in this study employs deep learning models, feature fusion, and optimal feature selection. Pretrained deep learning models, such as Xception, SqueezeNet, ShuffleNet, ResNet-18, ResNet-50, ResNet-101, NASNet-Mobile, MobileNet-v2, Inception-v3, Inception-ResNet-v2, GoogLeNet, GoogLeNet365, EfficientNet-b0, DenseNet-201, DarkNet-53, and DarkNet-19, were used for feature extraction. However, the extracted features exhibited low classification performance for the binary class problem (Table 2). Therefore, the deep features of the best pretrained models (ResNet-101 and EfficientNet-b0) were fused using a canonical correlation feature fusion approach, resulting in significantly better classification performance. The use of wrapper-based approaches for optimal feature selection guarantees better classification performance because the features are tested using a machine learning model. 

The b-IHHO wrapper-based approach was applied to remove redundant features and enhance the classification performance. The results demonstrated that the proposed framework features a high classification accuracy of 97.78 ± 0.33 (average ± standard deviation). The ability of b-IHHO to select more valuable features for classifying histopathological images owes to its effective search strategy. The conventional HHO only uses the objective function to select the features, which leads to a subpar classification performance. Therefore, the b-IHHO employed in this study uses three advanced search strategies in conjunction with an objective function, as discussed in Section 2.3.5. This enables quicker execution and higher accuracy for feature selection tasks, while reducing temporal complexity. Table 3 compares the classification accuracies of the proposed framework and other SOTA approaches.

Achieving a high OSCC detection accuracy has a tremendous significance and far-reaching implications, particularly for the early diagnosis of the disease. Timely and highly accurate OSCC detection using the proposed framework may significantly improve the prognosis and reduce mortality rates. Moreover, automatic histopathological image analysis may help practitioners maximize their workflow efficiency and enhance the diagnostic precision of OSCC detection.

## 5. Conclusions

This paper presented an automated OSCC detection framework that uses histopathological images for OSCC classification. First, various pretrained deep learning models were used to extract the deep features. The ResNet-101 and EfficientNet-b0 models yielded the highest accuracies of 91.51 and 91.61%, respectively, with 2048 and 1280 feature vector sizes, respectively. Subsequently, canonical correlation feature fusion analysis was conducted to concatenate the features, and an accuracy of 92.62% was achieved with a feature vector size of 2560. Moreover, the wrapper-based approach b-IHHO was used for feature selection and yielded the highest accuracy of 98.28%, with only 899 features. Additionally, comparisons with other wrapper-based feature selection approaches showed that the results of b-IHHO were statistically more stable, reliable, and significant (*p* < 0.01). Finally, a comparison with other SOTA methods also demonstrated the superiority and high classification performance of the proposed automated OSCC detection approach. 

## Figures and Tables

**Table 1:** Details and sample images in the OSCC biopsy datasets employed in this study.

	Normal	Sick (OSCC)
Histopathological images	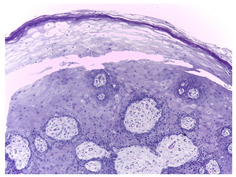	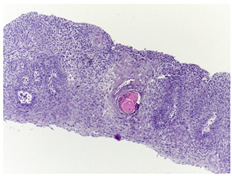
Images per class	2435	2511

**Table 2:** Classification performances of various pretrained models for OSCC detection.

Model	Feature Vector Size	Accuracy (%)
Xception	2048	87.77
SqueezeNet	1000	82.91
ShuffleNet	544	84.93
ResNet-18	512	86.05
ResNet-50	2048	89.69
ResNet-101	2048	91.51
NASNet-Mobile	1056	84.73
MobileNet-v2	1280	84.53
Inception-v3	2048	86.86
Inception-ResNet-v2	1536	89.69
GoogLeNet	1024	81.60
GoogLeNet365	1024	85.04
EfficientNet-b0	1280	91.61
DenseNet-201	1920	87.87
DarkNet-53	1024	86.96
DarkNet-19	1000	87.36

**Table 3:** Classification accuracies of the proposed framework and other SOTA approaches.

Study	Accuracy (%)
Sukegawa et al. [30]	86.22
Khan et al. [20]	92
Yu et al. [31]	92.78
Chang et al. [32]	92.81
Panigrahi et al. [33]	96.6
Yang et al. [34]	92.52
Das et al. [19]	97.82
This study	98.28 (mean = 97.78)

## Data Availability

The original data presented in the study are openly available in Kaggle at https://www.kaggle.com/datasets/ashenafifasilkebede/dataset, accessed 13 July 2024.
